# Differential methylation is associated with non-syndromic cleft lip and palate and contributes to penetrance effects

**DOI:** 10.1038/s41598-017-02721-0

**Published:** 2017-05-26

**Authors:** Lucas Alvizi, Xiayi Ke, Luciano Abreu Brito, Rimante Seselgyte, Gudrun E. Moore, Philip Stanier, Maria Rita Passos-Bueno

**Affiliations:** 1 Centro de Pesquisas Sobre o Genoma Humano e Células-Tronco, Instituto de Biociências, Universidade de São Paulo, São Paulo, Brazil; 2 Genetics and Genomic Medicine, Institute of Child Health, University College of London, London, UK

## Abstract

Non-syndromic cleft lip and/or palate (NSCLP) is a common congenital malformation with a multifactorial model of inheritance. Although several at-risk alleles have been identified, they do not completely explain the high heritability. We postulate that epigenetic factors as DNA methylation might contribute to this missing heritability. Using a Methylome-wide association study in a Brazilian cohort (67 NSCLP, 59 controls), we found 578 methylation variable positions (MVPs) that were significantly associated with NSCLP. MVPs were enriched in regulatory and active regions of the genome and in pathways already implicated in craniofacial development. In an independent UK cohort (171 NSCLP, 177 controls), we replicated 4 out of 11 tested MVPs. We demonstrated a significant positive correlation between blood and lip tissue DNA methylation, indicating blood as a suitable tissue for NSCLP methylation studies. Next, we quantified *CDH1* promoter methylation levels in *CDH1* mutation-positive families, including penetrants, non-penetrants or non-carriers for NSCLP. We found methylation levels to be significantly higher in the penetrant individuals. Taken together, our results demonstrated the association of methylation at specific genomic locations as contributing factors to both non-familial and familial NSCLP and altered DNA methylation may be a second hit contributing to penetrance.

## Introduction

Craniofacial development is a tightly regulated event that requires expression of many genes at a precise space-temporal specificity. These processes are carefully orchestrated by several well-documented molecular pathways, including FGF, BMP, TGF-β, SHH and WNT signalling pathways. Regulation of these pathways is influenced by a complex interaction between genetic and environmental factors, such that any interference in the regulation of these pathways may lead to abnormal phenotypes affecting the face and cranium which includes non-syndromic forms of cleft lip and/or palate (NSCL/P)^1–3^. NSCL/P is one of the most common congenital malformations in humans, affecting 1:700 live-births worldwide^4^. In broad terms, NSCL/P is considered to follow a multifactorial model^5, 6^, which has been supported by heritability studies with a genetic contribution estimated to vary from 45% to 85% depending on the population^7^. Common and rare variants identified through genomic analysis have successfully revealed several at-risk cleft alleles in distinct populations^8–12^. However, as for many other common diseases, the total sum of known variants still only explains a small percentage of the genetic contribution and NSCL/P falls within the missing heritability category^12, 13^. Studies on the environmental contribution for NSCL/P have mostly involved epidemiology of maternal exposures to factors such as malnutrition, alcohol, tobacco, folate and anti-epileptic drugs^14–19^, often with little explanation as to how those agents might disrupt molecular and cellular mechanisms that ultimately lead to a clefting phenotype. One possible explanation is their effect on epigenetic mechanisms, particularly methylation of DNA or histones. The contribution of differential DNA methylation to disease states can be assessed using genome wide analysis of CpG dinucleotides and MethWAS has been successfully applied in disease such as diabetes, rheumatoid arthritis and schizophrenia^20–24^. In this study, we have investigated the epigenetic contribution to NSCLP by performing a MethWAS, first in a Brazilian cohort and followed by a replication study in an independent cohort from the UK. We also compare methylation levels detected in blood to those from matched lip tissues obtained during surgery, in order to investigate differences arising from alternative, and perhaps more developmentally relevant tissues. We have further investigated methylation differences in familial NSCLP displaying incomplete penetrance upon *CDH1* mutations in order to verify whether methylation differences correlate to phenotype penetrance.

## Results

### NSCLP presents a distinct methylation signature enriched in regions of open and active chromatin

**Figure 1:**
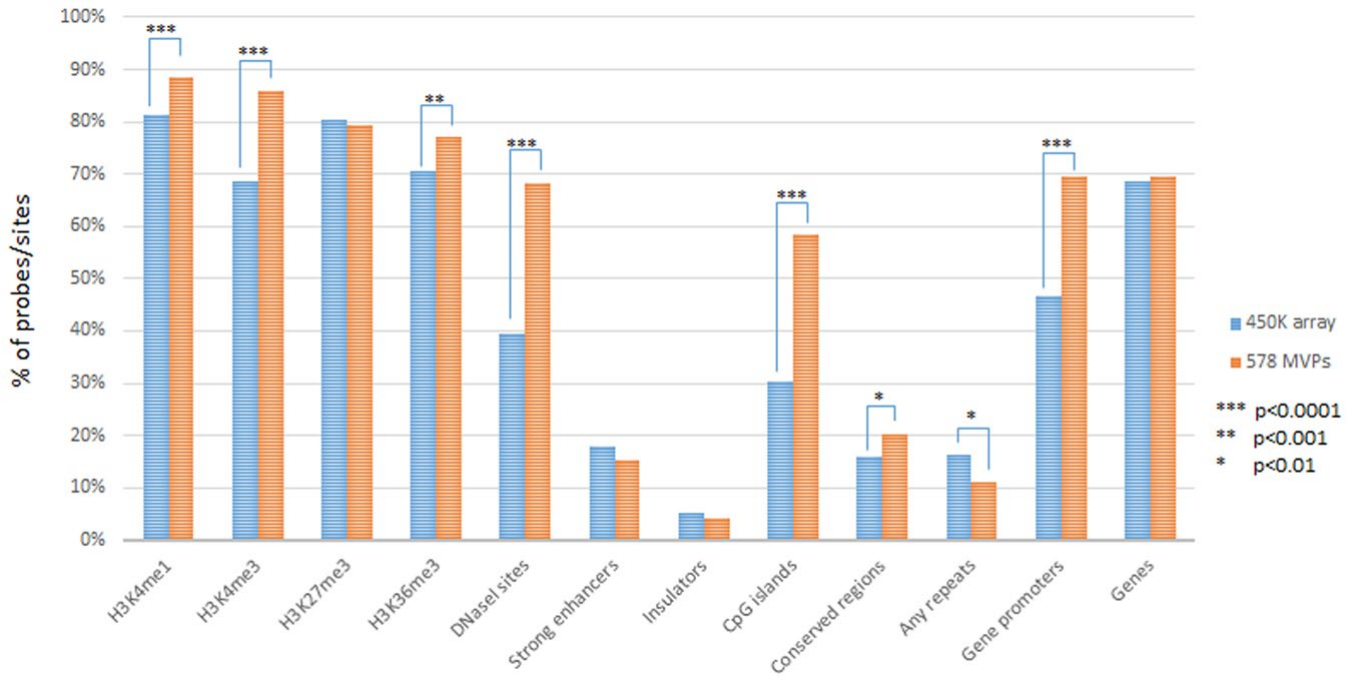
MVPs are enriched at active regions of the genome. Genomic distribution of EpiExplorer classes comparing the 578 MVPs and the Infinium Human Methylation 450 K Bead-Array (450 K array) filtered probes showing significant enrichment in the MVPs for active regions of the genome, including gene promoters (Chi-Square Test with Yates correction). Genomic coordinates from the 578 MVPs and 450 K filtered probes were used as input in the EpiExplorer online tool (http://epiexplorer.mpi-inf.mpg.de/) for genomic distribution and chromatin segments comparisons.

**Figure 2:**
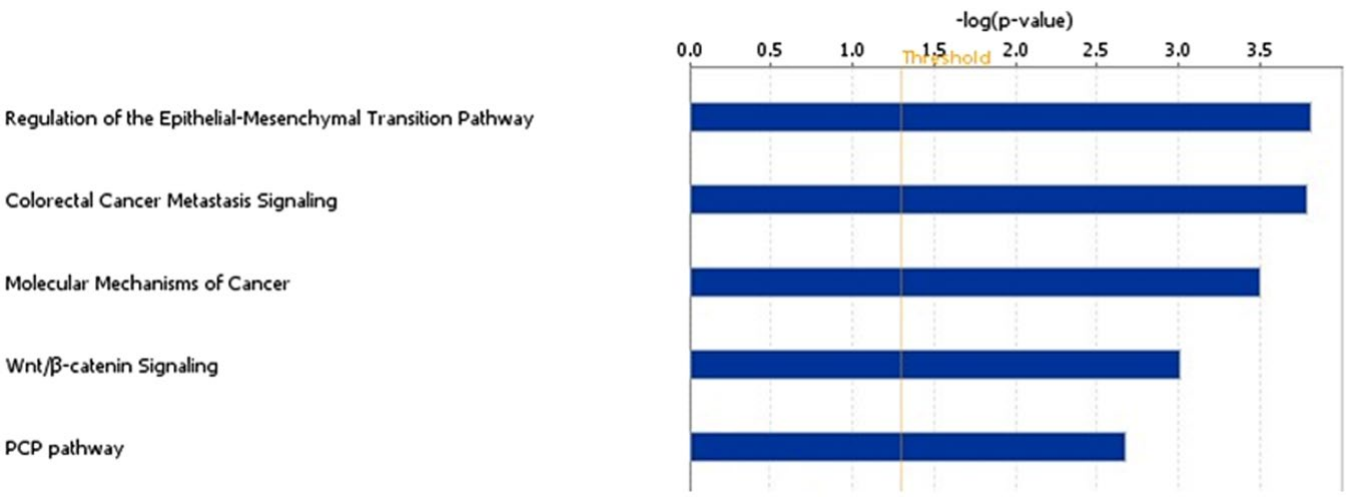
Enrichment of MVPs in canonical pathways related to craniofacial development. Top five canonical pathways on Ingenuity Pathway Analysis (IPA) using the 578 MVPs shows “Regulation of the Epithelial-Mesenchymal Transition Pathway”, “Wnt/B-catenin Signaling” and “PCP pathway” as significantly enriched in those MVPs. Those pathways are extensively related to craniofacial development, including lip and palate morphogenesis.

In this study, we therefore performed a MethWAS using a Brazilian age-matched cohort of 67 NSCLP and 59 control samples using the Infinium Human Methylation 450 K platform (Illumina). We found 578 MVPs at the genomic level of significance (p < 10^−7^, FDR adjusted) (Supplementary Table I), suggesting a different methylation signature in NSCLP samples. Next all 578 MVPs were subjected to an exploratory process in which a significant enrichment of MVPs was found for regions of open and active chromatin as marked by H3K4me1, H3K4me3, DNaseI sites and Gene Promoters (Fig. 1 and Supplementary Figure 1). Sixty-nine percent of MVPs belonged to promoters, in comparison to 47% from the 450 K array, which represented a significant enrichment (p < 0,0001, Chi-square test with Yates correction). Co-methylation of the identified MVPs was verified by comparing to their neighbouring CpG sites in a 500 bp window, both up and downstream. Sixty percent of these CpGs had the same methylation as the MVPs (Fisher’s exact test, p = 0.0043). When selected MVPs with methylation differences greater than 7% were chosen, the co-methylation rate was increased to 70%. Next MVPs belonging to genomic regions and genes that either associated with NSCLP or were involved in some aspect of craniofacial development were analysed (list from Jugessur *et al*.^25^). Eighteen MVPs were found in candidate genes or regions for CL/P representing a significant enrichment in clefting-associated regions (Fisher’s exact test, p = 0.00044). As a final step of the exploratory analysis, Ingenuity Pathway Analysis (IPA, QIAGen) was used to look for enriched canonical pathways in the 578 MVPs. Among the 5 top-ranked canonical pathways were the “Regulation of the Epithelial-Mesenchymal Transition Pathway” and “WNT Beta-Catenin Signaling” (Fig. 2). Both pathways are strongly associated with craniofacial development and also CL/P etiology^26–30^. Altogether, these results suggested the robustness of our data, and encouraged us to move forward with a validation step.

### Methylation differences replicates in an independent cohort

To validate the MethWAS findings, a replication study was carried out using an independent cohort of individuals with non-familial NSCLP recruited in the London area in the UK, which represented an ethnically and environmentally distinct population. Selection of candidate MVPs was based on the following combined criteria: the MVP with the lowest p-value and highest methylation difference (*C11orf58* MVP, probe ID cg10633981), two MVPs with high methylation differences (*chr1* MVP, probe ID cg15897635 and *chr17* MVP, probe ID cg09319020) and eight MVPs in candidate regions/genes for NSCLP (*FAT1* MVP, probe ID cg00405769; *FGF8* MVP, probe ID cg11706469; *FGFR1* MVP, probe ID cg20913106; *MYC* MVP, probe ID cg00611675; *PVRL1* MVP, probe ID cg06391300; *WHSC1* MVP, probe ID cg03150409; *WNT2B MVP*, probe ID cg11806528 and *WNT7A* MVP, probe ID cg13602813. In total, 11 MVPs were selected for replication in 171 NSCLP and 177 age and ethnicity-matched controls samples using a Bisulfite Amplicon Sequencing (BSAS) approach. In the replication cohort, significant differential methylation was found for 4 out of the 11 candidate MVPs (*chr1* MVP p = 0.03; *FAT1* MVP p = 0.0002; *MYC* MVP p < 0.0001; *WHSC1* MVP p = 0.04) (Table 1). MVPs at *MYC* and *FAT1* in the replication cohort were detected in the same direction of those in the MethWAS cohort, while the *chr1* and *WHSC1* MVPs presented in the opposite methylation direction than in the methylome analysis. All mean methylation values in the case group (NSCLP) were lower than those in the control group, even in non-significant MVPs, suggesting an apparent hypomethylation in the NSCLP group. In order to test if the overall methylation in the NSCLP group compared to controls was significantly reduced, methylation values from all CpG sites in the 11 analysed amplicons covered by the BSAS were investigated. No significant differences were found between methylation means from all tested amplicons (p = 0.7131, mean in NSCLP = 0.3377 ± 0.008810; mean in controls = 0.3423 ± 0.008716) (Supplementary Figure 2). Our results suggest that differential methylation is specific at those tested MVPs and not a generalised hypomethylation in those amplicons in the UK NSCLP cohort.

**Table 1:** Methylation differences for the 11 tested MVPs in the methWAS (Brazilian cohort, NSCLP N = 68, control N = 59) and Replication study (British cohort, NSCLP N = 171, control N = 177).

	450 K ID	Genomic coordinate	methWAS		Replication study			
			Meth Diff	adjusted p-value	Mean Methylation NSCLP	Mean Methylation Control	Meth Diff	p-value
FAT1 MVP	cg00405769	chr4:187539852	−0,075	4,66E-09	0.9244 ± 0.002172	0.9331 ± 0.0007216	−0,00871	0,0002
MYC MVP	cg00611675	chr8: 128748464	−0,02626	1,52E-09	0.003672 ± 0.0003027	0.009375 ± 0.0008236	−0,0057	< 0,0001
WHSC1 MVP	cg03150409	chr4: 1892317	0,07564	1,02E-08	0.3055 ± 0.009068	0.3302 ± 0.008440	−0,02473	0,04
PVRL1 MVP	cg06391300	chr11: 119600292	−0,0419	3,01E-13	0.001697 ± 0.0001290	0.001795 ± 0.0001241	−0,0001	0,58
chr17 MVP	cg09319020	chr17: 7304467	−0,11844	2,05E-09	0.8406 ± 0.001630	0.8428 ± 0.0007958	−0,00216	0,23
C11orf58 MVP	cg10633981	chr11: 16779768	0,12801	1,85E-16	0.8587 ± 0.005899	0.8683 ± 0.007230	−0,00963	0,3
FGF8 MVP	cg11706469	chr10:103535362	−0,04486	0,000000064	0.007407 ± 0.0002551	0.007412 ± 0.0002048	−0,00001	0,98
WNT2B MVP	cg11806528	chr1:113051977	0,0321	4,58E-08	0.005564 ± 0.0001993	0.005658 ± 0.0003072	−0,00009	0,79
WNT7A MVP	cg13602813	chr3:13920840	−0,03251	9,88E-08	0.01223 ± 0.0004003	0.01312 ± 0.0004133	−0,00088	0,12
chr1 MVP	cg15897635	chr1:220697615	0,10364	1,82E-14	0.7349 ± 0.003714	0.7450 ± 0.003034	−0,0101	0,03
FGFR1 MVP	cg20913106	chr8:38324522	0,0243	2,95E-12	0.004360 ± 0.0002625	0.004482 ± 0.0002628	−0,00012	0,74

We found significant methylation difference in the replication study for four MVPs (*FAT1* MVP, *MYC* MVP, *WHSC1* MVP and chr1 MVP). Considering direction of methylation, only *FAT1* MVP and *MYC* MVP were replicated with the same pattern as observed in the methWAS. (Genomic coordinates on hg18; Meth Diff: methylation difference).

### Methylation in blood correlates to methylation in lip tissue DNA

**Figure 3:**
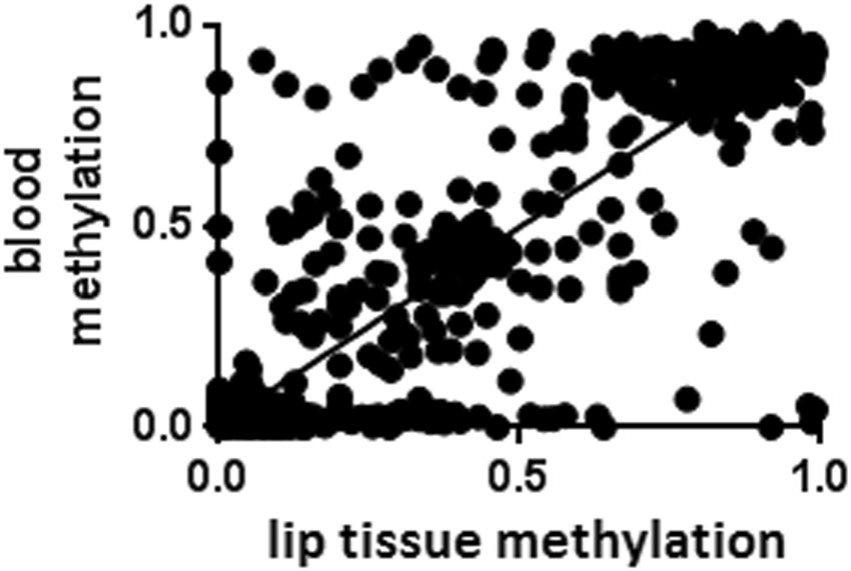
Whole-blood DNA methylation correlates to lip tissue DNA methylation. Linear regression between methylation values from all analysed MVPs and flanking regions covered by BSAS showing significant correlation between whole-blood and lip tissue methylation levels (R-square = 0.9028, p < 0.0001).

Both the MethWAS and the replication study were performed using whole-blood DNA. Clearly blood is not closely representative of the tissue types directly affected by the clefting phenotype during craniofacial development. However, it is not possible to obtain tissue samples at the time of lip formation from affected individuals. Acknowledging these deficiencies, we instead collected lip tissue samples from the time of first cleft repair surgery (n = 18) as the best available alternative. To examine possible tissue specific variability, we tested how well blood and lip tissues correlated for methylation findings. BSAS methylation values from the 11 tested amplicons were obtained in paired lip tissue samples and whole-blood DNA from 18 individuals. A linear regression analysis was performed to evaluate the correlation between the two sample types. This resulted in high and significant similarity (R-square = 0.9028, p < 0.0001) (Fig. 3).

### Promoter hypermethylation correlates to penetrance in familial NSCLP

**Figure 4:**
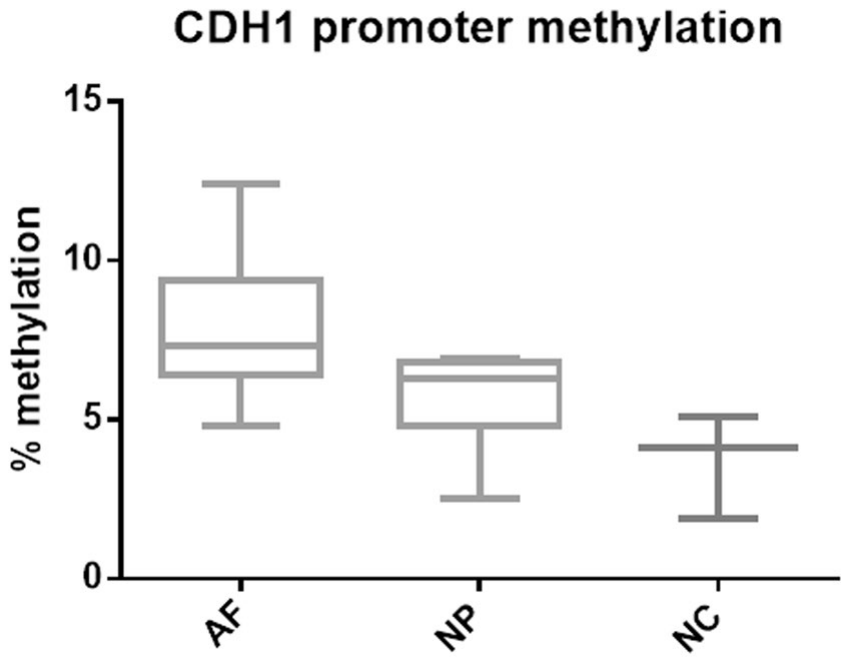
*CDH1* promoter methylation correlates to penetrance. Boxplot of *CDH1* promoter methylation levels in familial NS CL/P segregating *CDH1* mutations. Affected individuals (AF, n = 8) exhibit significantly higher *CDH1* promoter methylation levels in comparison to non-penetrant (NP, n = 7) and non-carriers (NC, n = 3) (p = 0,0112, Kruskal-Wallis test). We quantified methylation levels from 33 CpGs at *CDH1* promoter using conventional bisulfite sequencing in which 32 clones were analysed per sample.

We have recently shown that missense pathogenic mutations in *CDH1* play a role in the aetiology of NSCLP, particularly familial cases. However, *CDH1* mutations alone do not seem to be the only causative factor, as incomplete penetrance (~60%) was observed in these families^31^. We, therefore, hypothesised whether methylation could represent a second hit. Methylation levels of 33 CpG sites at the *CDH1* promoter in penetrant (n = 8), non-penetrant (n = 7) and non-carrier individuals (n = 3) were quantified using targeted bisulfite sequencing. *CDH1* promoter methylation was found to be higher in penetrant individuals than in non-penetrant and non-carriers (p = 0,0112, Kruskal-Wallis test, Fig. 4), suggesting methylation as a possible second hit to explain the cleft phenotype.

## Discussion

We hypothesised that NSCLP has a different epigenetic signature compared to controls. Indeed, after correcting our data for batch and confounding effects, our preliminary findings showed that we could detect significant enrichment of MVPs, which mostly lie in potential regulatory and active regions of the genome. We were also able to demonstrate a co-methylation pattern within our data which is compatible with methylation variation reported elsewhere^32^. Also, we showed that our MVPs were significantly enriched at NSCLP candidate regions and in canonical pathways reported to have relevance for the malformation such as WNT-β-catenin signalling and Regulation of the Epithelial-Mesenchymal-Transition pathway^26–30^. These results suggested the robustness of our data, and encouraged us to move forward with a validation step.

Replication of the significant methylation differences (in the same direction of difference) for 2 MVPs (*FAT1* and *MYC*) strongly suggests that methylation at those sites may well have importance for NSCLP in both populations. Indeed, the *MYC* locus represents a very strong candidate region, since it has been replicated by several NSCLP GWASs^9, 10, 33^, including patients from the Brazilian population^12^. The SNP at 8q24.21 region lies in an enhancer for *Myc* where disruption of the syntenic region in a murine model has been shown to result in clefts^34^. The *MYC* MVP we studied is located at the *MYC* promoter, which may potentially interfere with *MYC* expression levels. With respect to the *FAT1* locus, this gene encodes an atypical cadherin and is associated with the planar-cell-polarity pathway (PCP), which has been suggested to be important for craniofacial development^35, 36^. Differential methylation was also found for 2 candidate MVPs that were not in the same methylation direction as in the initial study (*chr1 MVP* and *WHSC1 MVP*). The chr1 MVP, selected by its large methylation difference in the Brazilian cohort, falls into a gene desert (1q41). We do not know if this is a regulatory region for genes in the vicinity, however, the closest gene *MARK1*, is involved in microtubule array and regulation of cell polarity and cell shape^37^. At the *WHSC1* MVP is located the *WHSC1* promoter for the Wolf-Hirschhorn syndrome candidate 1 gene. Interestingly this syndrome is characterised by multiple closure defects including cleft lip and cleft palate (OMIM#194190). Despite the inversion in the methylation direction, general methylation disturbances at those loci are possible mechanisms and either hypo- or hypermethylation could contribute to the phenotype. Therefore, we were able to replicate 36% of the tested MVPs in the UK cohort, despite the fact that these two populations are ethnically different and exposed to distinct environments. Taking into account the large social differences and potential genetic background variation between Brazil and the UK, we considered the validation of those MVPs as a high rate of concordance, which suggests a potential role for those methylation sites in the NSCLP etiology. Our results also suggest that there might be either shared environmental factors in these two populations or even distinct environmental factors leading to common epigenetic changes. In this regard, we were able to compare our 578 MVPs with 2965 sites differentially methylated associated to smoking during pregnancy^38^ and found two MVPs in common (cg14087168 and cg26015973), suggesting smoking as a contributing but not major environmental factor for our findings.

We also showed DNA methylation correlating to NSCLP penetrance in families displaying *CDH1* mutations. This would be the expected mechanism based on previous studies in *CDH1* mutated gastric cancer where abnormal cell behaviour leading to cancer depends on a two-hit model. In this case, the first hit is a pathogenic mutation present on one allele while the second hit is hypermethylation of the other allele^39, 40^. The same phenomena is true, for example, in the A/WySn mouse strain which exhibits spontaneous cleft lip/palate in 15% of neonates in which the *Wnt9b* gene is mutated on one allele and methylation of the other acts as a metastable epiallele, thus leading to the clefting phenotype^41^. Epigenetic mechanisms therefore may well play a role in the clinical expression of NSCLP for both familial and non-familial NSCLP.

It is notable that we could detect significant methylation differences both in non-familial and familial cases, despite investigating tissues from a different cell lineage from the malformation. It is possible that such differences may have been more pronounced in biologically relevant lip or palatal tissue DNA. Moreover, as we are studying a developmental defect from early gestation using postnatal tissues, we also realise that methylation differences and levels might not accurately reflect the relevant tissues at the time of lip and palatal formation. However, the methylation differences identified here might remain as a mark that occurred during development. Indeed, the biological significance of our findings are supported by the high correlation between methylation sites in blood and lip tissue DNA, and suggest that blood can serve as a proxy tissue for methylation studies involving craniofacial malformations.

In the longer term, it will be very important to better understand the function of the detected methylation differences. We can speculate that one possible effect might be to confer subtle differences on gene expression, which contributes to the phenotype. However, since mRNA is not available from the relevant samples, it is not possible in this study to examine for a potential correlation between mRNA levels and DNA methylation. Therefore, changes at the detected MVPs should be considered a marker of NSCLP, requiring further exploration to determine a direct or indirect role in the pathogenesis.

Although there are various epigenetic mechanisms known to be involved in gene regulation and disease, in this study we chose to analyse DNA methylation due to its well-known effect and the availability of suitable platforms previously developed for this approach. However, we acknowledge that other epigenetic mechanisms such as histone modifications could also be involved in NSCLP aetiology. Nevertheless, our approach also makes a good starting point because of the known correlation between DNA methylation and some histone marks across the human genome, especially in unmethylated regions of DNA, which tend to be enriched for acetylated histones whereas methylated regions tend to lack acetylated histones and correlate to H3K9 methylation^42, 43^. This is supported by evidence that certain environmental factors that have previously been associated with NSCLP, have also been independently reported to interfere with histone acetylation, methylation and phosphorylation^44–47^. Therefore, in the future it will be interesting to examine if the methylation differences we report here may also indicate alterations in histone modifications within the same regions.

In summary, we found a number of differentially methylated sites associated with NSCLP, providing evidence that epigenetic factors may play a role in the aetiology of this malformation. We also provide evidence that DNA methylation may represent a second hit in individuals with *CDH1* mutations. Further studies investigating how environmental factors interfere with the normal methylation pattern at these and other sites will be important. Investigation of how different methylation levels might functionally contribute to the clefting phenotype, such as misregulation of normal gene expression, will be required to explain our findings and to provide therapeutic targets in the future.

## Material and Methods

### Ethics

This study was approved by the Ethics Committee of the Instituto de Biociências (Universidade de São Paulo, Brazil) and Great Ormond Street Hospital for Children NHS Trust Ethics Committee. Biological samples were collected after signed informed consent by the parents or legal guardians. All experiments were performed in accordance with relevant guidelines and regulations.

### Patients and controls

The MethWAS cohort included 126 Brazilian samples, in which 67 were cases from non-familial NSCLP individuals (males = 37, females = 30; average age at sampling = 5.29 ± 0.53 yrs) and 59 age and sex-matched controls from healthy individuals (males = 28, females = 31, average age at sampling = 6.45 ± 0.51 yrs). Brazilian samples were ascertained either at the Hospital das Clínicas of Universidade de São Paulo (São Paulo, Brazil), Centro de Pesquisas Sobre o Genoma Humano e Células-Tronco of Universidade de São Paulo (São Paulo, Brazil) or during missions of Operation Smile Brazil, in the Brazilian state of Ceará. The replication cohort was composed by 348 samples from the UK, in which 171 were non-familial NSCLP (males = 107, females = 64; average age at sampling = 3.30 ± 0.46 yrs) and 177 age and sex-matched non-cleft controls with no family history of CLP (males = 100, females = 77; average age at sampling = 3.96 ± 0.01 yrs) recruited from the North East Thames Regional Genetics Service. Populations from both cohorts are genetically admixed, in which applying the concept of race is difficult. However, both populations are largely enriched for white European ancestry (~70%). Whole-blood DNA was extracted from all samples by the North Thames Regional Genetics Service. In addition to blood, lip tissue samples recovered as discard material at time of the first corrective surgery were stored in RNAlater (Thermo Scientific, USA) and available for 18 of these patients. DNA and RNA were extracted from these tissues using TRIzol Reagent (Thermo Scientific, USA) following the manufacturer’s protocol.

### MethWAS analysis

Samples from the MethWAS cohort (Brazilian cohort) were subjected to bisulfite conversion using 1ug of DNA in the EpitectBisulfite Kit (QIAGen). The samples were analysed on the Illumina HumanMethylation 450 K Bead-Array platform (Illumina) according to the recommendations at the sequencing and array facility DeoxiBiotecnologia (Araçatuba, Brazil). Because samples were split into 3 batches for hybridization, we re-submitted 12 samples (6 NSCLP, 6 controls) (here called as re-batch samples) to identify and correct for batch-effects between runs. Analysis consisted of two major steps: first to identify batch-effect markers and a second for differential methylation analysis. In the first step we compared the 12 paired samples, initial batch versus re-batch, using a t-test aiming to find differentially methylated sites. As the same sample was compared in two different batches, methylation differences could be assumed to be due to either batch or confounding effects. Selecting differentially methylated sites at the genomic level (t-test, p < 10^−7^), 270 sites were identified and these sites were then used as batch-effect markers for data correction. Using an interactive ranking method, which selected the lowest p-values among those 270 batch-effect markers, loci independent effects were identified in a total of 5 sites (cg20154084, cg00196012, cg23948080, cg22345647, cg09307868). When these sites were subsequently used as covariates for p-value correction, none of the remaining 265 sites showed significant p-values. Therefore these 5 sites were chosen as batch-effect markers and used as covariates for the next analysis step. The second step consisted of filtering, normalisation and differential methylation analysis using the RnBeads pipeline^48^. We filtered out probes affected by SNPs, on sex chromosomes, probes with a p-value detection >0.05 (Greedycut), probes with non-CpG methylation pattern and probes from sites with a p-value > 0.01 in the paired batch analysis. Data was normalised using the SWAN method. Differential methylation analysis was performed using the RefFreeEWAS method implemented in the RnBeads pipeline, which is a LIMMA based method and corrects for blood cellular heterogeneity. We also used sex, age and the 5 batch-effect markers as covariates for differential methylation analysis correction. We selected as MVPs those probes with differential methylation p-value < 10^−7^ after FDR adjustment.

### Investigation of MVPs in a replication cohort

To quantify methylation at candidates MVPs for replication, we used the Bisulfite Amplicon Sequencing (BSAS) method, which relies on bisulfite PCR, library preparation and DNA sequencing with a NGS sequencer^49^. In total, we selected 11 MVPs and designed bisulfite-specific PCR primers using the online tool MethPrimer (http://www.urogene.org/methprimer/). Primers sequences are available under request. Samples from the replication cohort and palatal tissue sample DNAs were submitted for bisulfite conversion using 2 ug of DNA in the e EZ-96 Methylation Kit (Zymo Research). Converted DNA was used as a template for bisulfite-specific PCR for each primer pair with the HotStartTaq Plus (QIAGen) standard protocol and products were checked by agarose gel electrophoresis. All 11 products were pooled per sample and sizes were checked using a DNA TapeStation prior to library preparation. During the library preparation indexes were added in one PCR step for each pooled sample (Access Array Barcode Library, Fluidigm). Libraries were purified by Ampure XP Beads in a magnetic column and checked again in the DNA TapeStation for peak shift visualization. Finally libraries were submitted for sequencing with the MiSeq Reagent V2 Kit 250 bp pair-endedrun on a MiSeq Sequencer (Illumina). We performed de-multiplexing of sequences using the FASTX Barcode Splitter program in the FastX Toolkit R package (http://hannonlab.cshl.edu/fastx_toolkit/). Following this, we filtered out reads of low quality, selecting only reads with at least 50% of bases with Q > 30 using the FASTQ Quality Filter program, also part of the FastX Toolkit R package. Next FASTQ files were converted to FASTA files using the FASTQ-to-FASTA program in the same package. For the quantification of methylation levels in the 11 candidate MVPs we used the BiQAnalyzer HT software^50^. The BiQAnalyzer HT also has some additional quality filters including sequence identity, conversion rate and gaps allowed in CpG sites. In this case we set the parameters to a minimal reference sequence identity to 90%, a minimal bisulfite conversion rate of 90% and a maximum of 10% gaps allowed in CpG sites. In order to achieve a high sensitivity, we also accepted samples with a minimum of 1000 reads after all filtering steps. Following these parameters, analysis was conducted per gene-region in all samples and we performed a comparison of methylation levels in each MVP site between NSCLP and control groups using an unpaired two-tailed t-test with Welch’s correction, for differential methylation analysis with level of accepted significance ≤0.05.

### Correlation of methylation in lip tissue and blood

DNA samples from lip tissue as well as whole blood were available from the same patients and were included in the BSAS work-flow. A linear regression analysis was performed to evaluate methylation differences between lip tissue and whole-blood at each of the tested sites (11 MVPs).

### CDH1 promoter methylation in familial NS CL/P

DNA samples from two families segregating CDH1 mutations with NS CL/P previously described by Brito *et al*.^31^ were used for CDH1 promoter methylation quantification by conventional bisulfite sequencing. Those samples included 8 affected individuals, 7 non-penetrant individuals and 3 non-carriers. DNAs were submitted to bisulfite conversion with the EpiTect Bisulfite Kit (QIAGen) and PCR using primers for the bisulfite converted CDH1 promoter, covering 33 CpGs. Amplicons were cloned using the TOPO Cloning Kit (ThermoFisher) and 32 clones per sample were submitted to Sanger Sequencing. Sequencing results were analysed with the online tool BISMA (http://services.ibc.uni-stuttgart.de/BDPC/BISMA/) and the total methylation values were compared among affected, non-penetrant and non-carriers groups using Kruskal-Wallis test. Level of significance accepted ≤0.05.

## Electronic supplementary material

Supplementary Figure 1 and 2

Supplementary Table I
