# Supplementary material for: Differential methylation is associated with non-syndromic cleft lip and palate and contributes to penetrance effects
Source: Sci Rep. 2017 May 26;7:2441. doi: 10.1038/s41598-017-02721-0 (PMC5446392; doi:10.1038/s41598-017-02721-0)
Supplement: Supplementary file 1 — Supplementary Figure 1 and 2 [file 41598_2017_2721_MOESM1_ESM.doc]

**Supplementary Figures**

***Differential methylation is associated with non-syndromic cleft lip and palate and contributes to penetrance effects***

*Lucas Alvizi^1^, Xiayi Ke^2^, Luciano Abreu Brito^1^, Rimante Seselgyte^2^, Gudrun E. Moore^2^, Philip Stanier^2,*^, Maria Rita Passos-Bueno^1,*^****.***

1- Centro de Pesquisas Sobre o Genoma Humano e Células-Tronco, Instituto de Biociências, Universidade de São Paulo, São Paulo, Brasil.

2- Genetics and Genomic Medicine, Institute of Child Health, University College of London, London, UK.

*Corresponding authors: [passos@ib.usp.br](mailto:passos@ib.usp.br) ; [p.stanier@ucl.ac.uk](mailto:p.stanier@ucl.ac.uk)


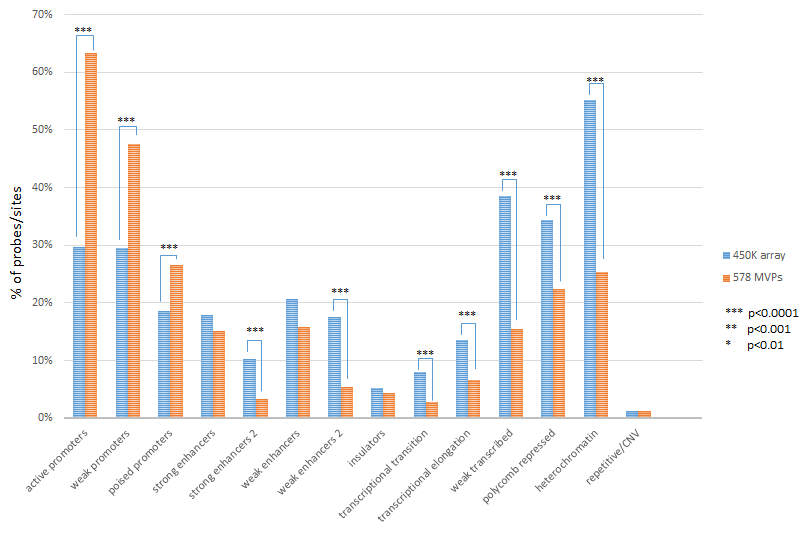
**Supplementary Figure 1: MVPs are enriched in regulatory regions of the genome**. Comparison of Chromatin segments from EpiExplorer between the 578 MVPs and the Infinium Human Methylation 450K Bead-Array (450K array) filtered probes showing significant enrichment in the MVPs for active regions as “active promoters”, “weak promoters”, “poised promoters”. Alternatively, regions as “weak transcribed”, “polycombrepressed” and “heterochromatin” are significantly less represented in our MVPs (Chi-Square Test with Yates correction).


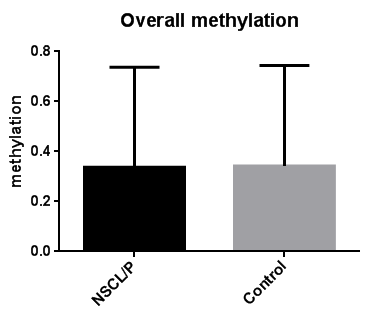


**Supplementary Figure 2**: **Comparison of overall methylation values between NSCLP and control groups showing no significant difference**. We used methylation values from all tested MVP and flanking regions covered by the BSAS and compared groups with a unpaired T-test (p= 0.7131).
